# A Phytoestrogen-Rich Diet Increases Energy Expenditure and Decreases Adiposity in Mice

**DOI:** 10.1289/ehp.10413

**Published:** 2007-07-12

**Authors:** Christopher R. Cederroth, Manlio Vinciguerra, Françoise Kühne, Rime Madani, Daniel R. Doerge, Theo J. Visser, Michelangelo Foti, Françoise Rohner-Jeanrenaud, Jean-Dominique Vassalli, Serge Nef

**Affiliations:** 1 Department of Genetic Medicine and Development and National Center for Competence in Research - Frontiers in Genetics, University of Geneva, Geneva, Switzerland; 2 Department of Cellular Physiology and Metabolism, Faculty of Medicine, University of Geneva, Geneva, Switzerland; 3 National Center for Toxicological Research, Jefferson, Arkansas, USA; 4 Department of Internal Medicine, Erasmus Medical Center, Rotterdam, the Netherlands; 5 Department of Internal Medicine, Faculty of Medicine, University of Geneva, Geneva, Switzerland

**Keywords:** AgRP, endocrine disruptors, isoflavones, lipid oxidation, obesity, phytoestrogens

## Abstract

**Background:**

Obesity is an increasingly prevalent health problem, and natural effective therapeutic approaches are required to prevent its occurrence. Phytoestrogens are plant-derived compounds with estrogenic activities; they can bind to both estrogen receptors α and β and mimic the action of estrogens on target organs.

**Objectives:**

The purpose of this study was to examine the influence of soy-derived phytoestrogens on energy balance and metabolism.

**Methods:**

Male outbred mice (CD-1) were allowed *ad libitum* access to either a high soy-containing diet or a soy-free diet from conception to adulthood. We measured circulating serum isoflavone levels using reverse-phase solid-phase extraction for subsequent liquid chromatography electrospray tandem mass spectrometry analysis. Adult animals were analyzed for body composition by dual-energy X-ray absorptiometry, locomotor activity by running-wheel experiments, respiratory exchange rate by indirect calorimetry, and food intake using metabolic cages. Quantitative reverse transcriptase-polymerase chain reaction was performed to determine the expression of hypothalamic neuropeptide genes.

**Results:**

We found that adult mice fed a soy-rich diet had reduced body weight, adiposity, and resistance to cold. This lean phenotype was associated with an increase in lipid oxidation due to a preferential use of lipids as fuel source and an increase in locomotor activity. The modulation of energy balance was associated with a central effect of phytoestrogens on the expression of hypothalamic neuropeptides, including agouti-related protein.

**Conclusion:**

The data suggest that dietary soy could have beneficial effects on obesity, but they also emphasize the importance of monitoring the phytoestrogen content of diets as a parameter of variability in animal experiments.

In Western society obesity has become a major health problem, and it is beginning to replace undernutrition and infectious diseases as the most significant contributor to ill health worldwide. Overweight individuals have a strong predisposition to type 2 diabetes, hyperlipidemia, hypercholesterolemia, and hypertension (Kopelman 2000). We still do not fully understand the multiple factors involved in the etiology of diabetes and obesity or how to prevent their development. This is particularly unfortunate because these conditions are treated by diet and exercice with only mixed success. Dietary intervention to control excess body weight, hyperglycemia, and dyslipidemia have included low-energy and low-fat diets but are of limited efficacy and require strict and long-term commitment. Therefore, new diet-based therapies to prevent the occurrence of diabetes and obesity would be very valuable.

Estrogens are major regulators of lipid metabolism. They affect adipose tissue directly by regulating lipogenesis, lipolysis, or adipogenesis, or indirectly by modulating appetite or energy expenditure [for review see Cooke and Naaz (2004)]. In humans, estrogen insufficiency is responsible for the increase in adiposity during menopause. Indeed, post-menopausal women treated with estrogen replacement therapies do not exhibit the characteristic abdominal weight-gain pattern associated with menopause (Gambacciani et al. 1997). Similarly, the importance of estrogens in lipid metabolism is highlighted by the fact that individuals with mutations in the aromatase gene exhibit truncal obesity, insulin resistance, hypercholesterolemia, and hypertriglyceridemia (Carani et al. 1997). Further confirmation of the role of estrogens in regulating lipid metabolism has been obtained in mice lacking estrogen receptor-α (*ER-*α) or the aromatase gene: Both display adipocyte hyperplasia and hypertrophy (Heine et al. 2000; Jones et al. 2000). Recently, specific silencing of ER-α in the hypothalamus of female rodents has been shown to lead to obesity, hyperphagia, and reduced energy expenditure, indicating that ER-α in hypothalamic neurons plays an essential role in the control of energy balance and the maintenance of normal body weight (Musatov et al. 2007).

Phytoestrogens are nonsteroidal compounds that can bind to both ER-α and ER-β because of their ability to mimic the conformational structure of estradiol (Kuiper et al. 1997, 1998), and thereby imitate the actions of estrogens on target tissues (Barnes 1998). Phytoestrogens are found in many legumes and are particularly abundant in soy products. Genistin and daidzin, two major soy isoflavone glucosides, are present at high concentrations in soybeans and soybean-derived products and are a major source of xenoestrogen exposure in both humans (e.g., soy-based formula for infants, tofu) and animals (most commercially available diets). Certain Asian populations may consume up to 1 mg/kg of body weight per day, and infants fed soy-based formula ingest even higher amounts of isoflavones on a per-weight basis (Setchell et al. 1997). Multiple studies in humans and animals suggest that, similar to estrogens, dietary phytoestrogens play a beneficial role in reducing obesity and improving glucose control [for review see Bhathena and Velasquez (2002)]. Rodents fed phytoestrogens have significantly decreased adipose tissue compared with animals fed a phytoestrogen-free diet (Lephart et al. 2004a), and in humans, consumption of isoflavones is associated with lower body mass index and lower insulin levels (Goodman-Gruen and Kritz-Silverstein 2001). Similarly, feeding mice with pure dietary genistein at doses of 500–1,500 ppm has antilipogenic effects and decreases adipose deposition (Naaz et al. 2003). However, the physiologic mechanisms underlying the metabolic action of phytoestrogens on lipid metabolism and fat deposition have not yet been determined. The purpose of the present study was to characterize the influence of dietary soy-derived phytoestrogens on energy balance and adipose tissue. We show here that mice exposed to phytoestrogens maintain a lean phenotype associated with hyperphagia, and have increased locomotor activity, increased use of lipid as a fuel source, and decreased resistance to cold. The modulation of the energy balance is associated with altered expression of hypothalamic peptides involved in energy metabolism, including agouti-related protein (AgRP).

## Material and Methods

### Diets

CD-1 mice were maintained on *ad libitum* access to either a high soy–containing [high phytoestrogen (HP)] diet (Harlan Teklad 8604; Harlan Teklad, Madison, WI, USA], or a soy-free [low phytoestrogen (LP)] diet (Zeigler Phytoestrogen Reduced Rodent Diet I; Zeigler Brothers, Gardner, PA, USA). Weber et al. (2001) reported the isoflavone content of these two closed-formula diets to be approximately 150 ppm daidzein and 190 ppm genistein equivalents in the HP diet and nondetectable in the LP diet. These concentrations of isoflavones are consistent with a soy content of approximately 25% in the HP diet. Both diets were equivalent in terms of carbohydrate, protein, fat, amino acid, vitamin, and mineral content (Weber et al. 2001). In the LP diet formulation, soy was omitted and replaced by lactic casein and dried skim milk. The gross energy content (1,626 mJ/ 100 g for the HP diet versus 1,668 mJ/100 g for the LP diet), the metabolizable energy (3,100 vs. 3,240 kcal/kg), and the digestable energy (3,300 vs 3,530 kcal/kg) were similar for both diets.

### Animal care

CD-1 male and female mice were purchased from Charles River (Arbresle, France) for breeding. Animal protocols used in these studies were approved by the Commission d’Ethique de l’Expérimentation Animale of the University of Geneva Medical School and the Geneva Veterinarian Office. Animals were treated humanely and with regard for the alleviation of suffering. In short, male and female mice used for breeding were fed with the respective diets 6 weeks before mating so that the offspring of the pairing would be exposed solely to HP or LP diets. Male and female mice used in this study were therefore exposed to high or low levels of isoflavones from conception onward, with a broad window of exposure encompassing both embryonic and postnatal development as well as the adult period. Mice were housed in poly-styrene instead of polycarbonate cages to avoid potential contamination with bisphenol A, a well-known endocrine-disrupting compound with estrogenic activity.

### Plasma phytoestrogen levels

The concentrations of total genistein, daidzein, and equol were determined in individual plasma samples collected at 0800 hours from adult male mice (23–26 weeks of age) exposed throughout life to HP (*n* = 12) or LP (*n* = 12) diets. Enzymatically deconjugated plasma samples were processed using reverse-phase solid-phase extraction for subsequent liquid chromatography electrospray tandem mass spectrometry quantification using isotope dilution in conjunction with deuterium-labeled internal standards, as described previously by Twaddle et al. (2002). Method accuracies were determined to be 86–102% for spiked blank serum with relative SDs in the range of 0.5–3.3%.

### Body composition determination by DEXAscan

We used peripheral dual energy X-ray absorptiometry (DEXA; PIXImus; GE-Lunar Corp., Madison, WI, USA) to measure the *in vivo* percentage of fat mass of male and female mice.

### Food intake

We measured food intake in 3- and 6-month-old male mice (HP or LP) housed individually in metabolic cages with grid bottoms (3700M021; Indulab, Gams, Switzerland). For habituation, animals were maintained in metabolic cages for 48 consecutive hours every week for 3 weeks. In the fourth week, animals were transfered to metabolic cages, accustomed during the first day, and data were then collected for the remaining 24 hr.

### Rotarod and open-field test

Spontaneous locomotor activity was assessed using an open-field test (45 cm × 45 cm) with an LE 8811 motor activity monitor (Bioseb, Chaville, France). Male mice were tested for 10 min, and activity was monitored automatically with infrared beams. Data were recorded for each mouse every minute, then analyzed and stored using SeDaCom32 analyzer software (Bioseb).

To assess physical motor coordination, male mice were tested for their ability to stay on an accelerating rotarod (accelerating model; Ugo Basile, Comerio, Italy). After a 10-sec habituation period on the stationary rod, the mice were tested with the rod slowly accelerating at a constant pace from 4 to 40 rpm. The rotation speed was increased every 30 sec by 4 rpm, with a maximum test duration of 5 min. Mice were scored on their latency to fall from the rotating rod. Mice that fell within the first 10 sec were allowed one additional trial immediately after the first. In these few instances, the score from the second trial was used in subsequent data analysis. The tests were performed during two successive days with three trials per day and an intertrial interval of 20 min.

### Blood and tissue chemistry

Blood was collected by cardiac puncture from male mice that had fasted 2 hr in the morning. Sera and relevant peripheral tissues were stored at −20°C and used subsequently to assess the levels of metabolic hormones.

### Histology

Tissues were fixed in Bouin’s solution and embedded in paraffin. Sections (5 μm) were stained with hematoxylin and eosin (H&E). Images were captured and analysis was performed with SigmaScan (Jandel, San Rafael, CA, USA). We performed morphometric analysis on adipose tissues by measuring the surface of adipocytes in five representative sections (~ 1,100 mm^2^ each) for every HP and LP epididymal white adipose tissue (WAT). The data were collected and analyzed using the ImageJ software.

### Locomotor activity

Male mice (24–27 weeks of age) were housed individually in large plastic cages, each equipped with a running wheel, in ventilated, light-tight cabinets. Each wheel revolution was registered by a magnetic switch connected to a counter; the number of revolutions was recorded every minute for 8 consecutive days. The data were measured and analyzed using Chronobiology Kit computer software (Stanford Software Systems, Santa Cruz, CA, USA).

### Indirect calorimetry

Whole-body oxygen consumption rates, respiratory exchange rates, and metabolic rates were measured by indirect calorimetry using a four-chamber open-circuit Oxymax system (Colombus Instruments, Columbus, OH, USA). HP and LP male mice (25–28 weeks of age) were housed individually in plexiglas cages with an air flow of 0.6 L/min and a sample flow of 0.5 L/min at 23°C. After mice were given 2 days to adapt to the metabolic chamber, we measured the volume of oxygen consumed (VO_2_) and the volume of carbon dioxide produced (VCO_2_) at 15-min intervals for 48 hr. The metabolic rate (kilo-calories per hour) was calculated from the following equation: (3.815 + 1.232 × RER) × VO_2_, where RER is the respiratory exchange ratio (VCO_2_/VO_2_).

### Real-time RT-PCR (reverse transcriptase-polymerase chain reaction)

Total RNAs from male hypothalamus were extracted using the RNeasy mini kit (Qiagen, Hombrechtikon, Switzerland) according to the manufacturer’s protocols. RNA integrity and quantity were assessed using RNA 6000 nanochips with an Agilent 2100 bioanalyzer (Agilent Technologies, Palo Alto, CA, USA). One microgram of total RNA was reverse-transcribed with the Omniscript RT-kit (Qiagen) according to manufacturer’s instructions. For each PCR reaction, 1/20th of the cDNA template was PCR amplified in a 7900HT SDS System using Power SYBR Green PCR master mix (both from Applied Biosystems, Foster City, CA, USA). We obtained raw threshold-cycle (*Ct*) values using SDS 2.0 software (Applied Biosystems). Relative quantities (*RQ*) were calculated with the formula *RQ* = *E* − *Ct* using efficiencies (*E*) calculated for each run with the DART-PCR algorithm, as described by Peirson et al. (2003). A mean quantity was calculated from triplicate PCR reactions for each sample, and this quantity was normalized to the average of four endogenous control genes (cyclophilin B, α2-tubulin, β-tubulin, or β-actin) as described by Vandesompele et al. (2002). Normalized quantities were averaged for three replicates for each data point and presented as the mean ± SD. We arbitrarily designated the highest normalized relative quantity as a value of 1.0. Fold changes were calculated from the quotient of means of these normalized quantities and reported as ± SE. We determined the statistical significance of fold changes by paired Student’s *t*-test. The relevant primers used for PCR are listed in the Supplemental Material (available online at http://www.ehponline.org/members/2007/10413/suppl.pdf).

## Results

### Exposure to dietary phytoestrogens

**Figure 1:**
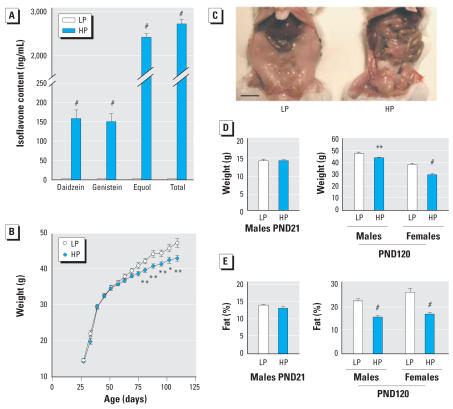
Leanness in mice exposed to soy in the diet. (*A*) Serum isoflavone levels of adult male mice fed HP or LP diet. (*B,C*) Body weight gain was significantly reduced in HP male mice; bar = 1 cm. Total weight (*D*) and fat content (*E*), as measured by DEXA analysis, were similar at weaning stage (postnatal day 21; PND21) but were significatively reduced in adult HP animals (PND120). Note that total weight and fat content were slightly more reduced in female than in male HP mice. Results are mean ± SE (*n* = 12/group, except for morphologic analysis where *n* = 5/group). * *p* < 0.05, ** *p* < 0.01, and ^#^*p* < 0.001 compared with control.

Male CD-1 mice were exposed to and fed either the HP diet or the LP diet from conception to adulthood. Weber et al. (2001) reported the isoflavone content of these two closed-formula diets to be approximately 150 ppm daidzein and 190 ppm genistein equivalents in the HP diet and nondetectable in the LP diet. The levels of isoflavones in plasma and diet were directly correlated (Figure 1A); the average steady-state plasma concentrations of genistein (160 ± 79 ng/mL) and daidzein (150 ± 73 ng/mL) in mice consuming the HP diet were well within the range observed in humans consuming soy foods and nutritional supplements (King and Bursill 1998; Setchell et al. 1997), whereas the daidzein metabolite equol was present at higher concentrations (2,400 ± 290 ng/mL) than those observed in humans. In contrast, the levels of plasma genistein, daidzein, and equol were undetectable (< 5 ng/mL) in mice fed the LP diet.

### Reduced weight and adipose tissues in mice exposed to phytoestrogens

Although body weights were similar at weaning, a significant difference developed progressively in adults so that at 4 months of age, male HP mice weighed 7.6% less than LP mice (Figure 1B). We found a 2% reduction in body length (naso-anal distance) in adults (LP, 10.37 ± 0.08 cm; HP, 10.12 ± 0.06 cm; *p* < 0.05; *n* = 23 in each group), suggesting that embryonic or postnatal growth were slightly affected by dietary phytoestrogens. Overall fat content was markedly lower in male HP mice (a 31% reduction at 4 months of age; Figure 1C), as determined by DEXAscan (Figure 1E). Interestingly, in females, reduction in weight (22%) and fat content (36%) were more pronounced compared with male HP mice (Figure 1D, E).

**Figure 2:**
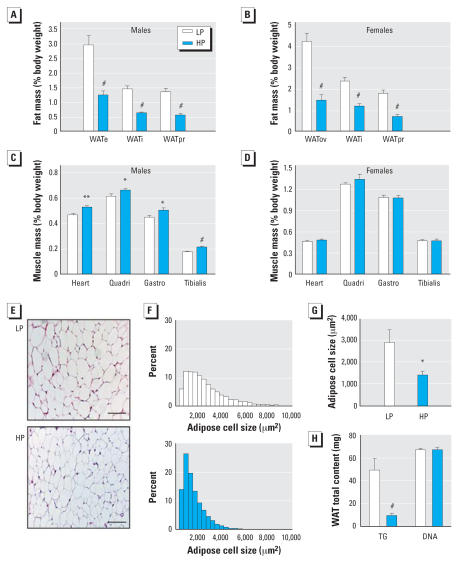
Adiposity is drastically reduced in animals fed the HP diet. Abbreviations: WATe, epididymal WAT; WATi, inguinal WAT; WATov, ovarian WAT; WATpr, perirenal WAT. Fat mass (% of body weight) was less pronounced in 5-month-old male HP mice (*A*) than in 4-month-old female mice (*B*). We also observed a significant increase of skeletal muscle mass in quadriceps (Quadri), gastrocnemius (Gastro), tibialis, and heart in males (*C*) but not in females (*D*). (*E*). H&E staining of representative epididymal WAT sections of 5-month-old HP (bottom) and LP (top) male mice; bar = 100 μm. The distribution (*F*) of cell size of LP (top) and HP (bottom) male mice reveals a significant reduction of the mean adipose cell surface (*G*). (*H*) Total triglyceride (TG) and DNA in the WATe of HP males. Results are mean ± SE (*n* = 12/ group). *p < 0.05, **p < 0.01, and ^#^*p* < 0.001 compared with control.

Anatomical observations revealed a striking difference in adipose tissue (Figure 1C). After normalization to body weight, fat depots such as the epididymal, inguinal, and peri-renal WAT were reduced by > 50% (Figure 2A,B) in contrast to other major organs such as liver and spleen, which were not affected (data not shown). We also observed a slight but significant increase in skeletal muscles and heart of male but not female mice exposed to the HP diet (Figure 2C,D). This increased muscular mass may reflect the increased locomotor activity observed in HP mice.

Sections of WAT showed decreased adipocyte size in HP mice (Figure 2E); exposure to phytoestrogens resulted in an average 2-fold reduction in the median cytoplasmic area (Figure 2F,G) of WAT and a 5-fold decrease in total triglyceride content (Figure 2H). In contrast, total DNA content was not altered (Figure 2H), indicating that decreased fat mass is exclusively the result of reduced fat storage in HP animals.

### Reduced thermogenic activity in HP mice

**Figure 3:**
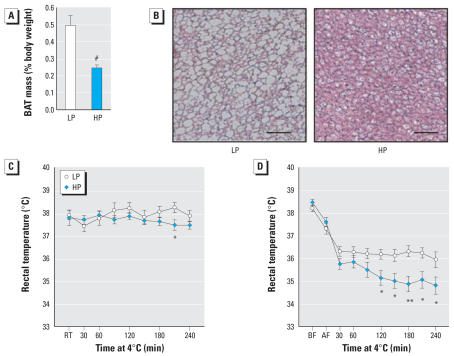
BAT weight and resistance to cold in male mice fed the HP or LP diets. Abbreviations: AF, after fasting; BF, before fasting; RT, room temperature. BAT weight (*A*) and histology of 5-month-old LP and HP mice (*B*; bar = 100 μm. Body temperature of adult HP and LP mice exposed 4°C for 4 hr BF (*C*) or AF 12 hr (*D*). Results are mean ± SE. *p < 0.05, **p < 0.01, and ^#^*p* < 0.001 compared with control.

Adaptive thermogenesis might also be altered in mice exposed to phytoestrogens. Interestingly, we found the interscapular brown fat depot to be 51% smaller in male mice fed the HP diet compared with those fed the LP diet (Figure 3A, *n* = 15 and 14, respectively; *p* = 0.002). Histologic analysis indicated that lipid droplets in HP brown adipocytes were much smaller than those of LP brown adipocytes (Figure 3B). Although the thermogenic capacity in mice depends on brown adipose tissue (BAT) activity, the massive reduction in BAT size and its lipid content in HP mice did not affect body temperature in normal conditions. At 20°C, HP and LP mice had similar body temperature (Figure 3C). To challenge the thermogenic capacity of these animals, we exposed them to cold at 4°C for a period of 4 hr. In the fed state, HP mice withstood the cold as well as the LP mice; however, in the fasted state (Figure 3D), the body temperature of HP mice fell much faster than that of the LP mice, indicating that dietary phytoestrogens affect resistance to cold. To assess how phytoestrogen exposure influences energy metabolism in brown adipocytes, we determined the mRNA expression of genes involved in lipid metabolism in BAT. We found that the reduction in BAT weight in HP mice was partially compensated by up-regulating uncoupling protein-1 (1.5-fold increase; *n* = 8–9/group; *p* < 0.05; data not shown) and down-regulating the lipogenic gene acetyl-CoA carboxylase-1 (4.2-fold decrease; *n* = 8–9/ group; *p* < 0.05; data not shown), two important genes regulating thermogenesis and lipid oxidation.

### HP mice are hyperphagic and exhibit increased metabolic rate, lipid utilization, and locomotor activity

**Figure 4:**
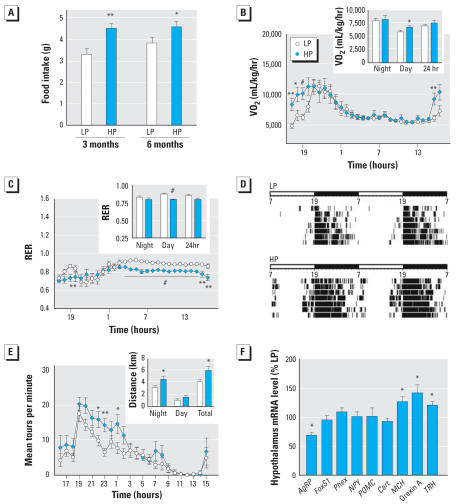
Food intake, lipid oxidation, and locomotor activity in male LP and HP mice. Abbreviations: FoxS1, forkhead transcription factor; Phex, phosphate-regulating gene with homologies to endopeptidases on the X chromosome. (*A*) Food intake measured at 3 and 6 months of age (*n* = 12/group). Metabolic rate (V0_2_; *B*) and RER in the resting period (*C*) determined by indirect calorimetry analysis. Locomotor activity was higher in the first part of the dark phase in adult HP mice compared with LP mice (*n* = 11–12/group) as represented by an actiogram (*D*) or as the average daily activity over a 24-hr period (*E*). Gene expression determined by real-time quantitative PCR in hypothalami (*F*) is shown for each gene as percent of expression in HP mice relative to LP animals (*n* = 10/group). Results are mean ± SE. *p < 0.05, **p < 0.01, and ^#^*p* < 0.001 versus control.

To determine whether the lean phenotype of HP mice could be a consequence of a difference in their total energy balance, we first assessed daily food intake using metabolic cages. Surprisingly, male mice maintained on an HP diet were hyperphagic, consuming 41% and 18% more food than LP-fed mice at 3 and 6 months of age, respectively (Figure 4A). Total energy expenditure, which includes physical activity, basal metabolism, and adaptive thermogenesis, should therefore also be altered in HP mice. Indeed, mice exposed to phytoestrogens exhibited a significant increase in metabolic rate (VO_2_; Figure 4B) and energy expenditure (data not shown), as well as a significant reduction in the RER (Figure 4C) as measured by indirect calorimetry. The RER reflects the ratio of carbohydrate to fatty acid oxidation; therefore, these results are consistent with HP mice using a greater ratio of lipids as a fuel source during the resting phase.

To assess whether differences in activity levels may also be involved, we subjected LP and HP mice to behavioral tests. Although HP and LP mice were indistinguishable in both rotarod and open field activity tests (data not shown), the nightly locomotor activity (measured by the running wheel) of HP mice was 45% higher (Figure 4D,E). As expected, interleukin-6 mRNA, whose production in skeletal muscle is markedly increased by exercise, was up-regulated in tibialis skeletal muscle of HP animals (2.03-fold increase by quantitative RT-PCR; *n* = 8–9/group; *p* < 0.05; data not shown). It remains possible that the improved locomotor performance might have been due to differences in body weight, but we view this as unlikely because we found no correlation between body weight and locomotor performance within groups (data not shown). Overall, increased energy expenditure and locomotor activity, coupled with a marked shift toward the use of lipids, must be important contributing factors for the leanness of HP mice.

### A central effect of phytoestrogens on the expression of hypothalamic neuropeptides

Estrogens are known to modulate locomotor activity in rodents (Ogawa et al. 2003). The increased locomotor activity of HP mice suggests a direct central effect. Indeed, we found that the abundance of *AgRP* gene transcripts was down-regulated by 32%, whereas orexin A, MCH (melanocortin hormone), and TRH (thyroid-releasing hormone) mRNAs were up-regulated by 40%, 25%, and 22%, respectively, in the hypothalamus of HP animals (Figure 4F). In contrast, *NPY* (neuropeptide Y)*, POMC* (proopiomelanocortin), and *Cart* (cocaine and amphetamine regulated transcript) gene expression were not affected. Increased production of orexin A and MCH, which are both orexigenic peptides, may explain the increased food intake observed in HP mice. Consistent with our data, *AgRP* gene expression has been shown to be repressed in clonal NPY/AgRP hypothalamic neurons exposed to 17β-estradiol (Titolo et al. 2006). As we found to be the case for phytoestrogen-exposed mice, aging *AgRP*-knockout animals are lean because of increased metabolic rate, lipid utilization, and locomotor activity (Wortley et al. 2005). Available evidence suggests that AgRP has central inhibitory action on the hypothalamic–pituitary–thyroid axis by suppressing hypothalamic TRH expression and thereby decreasing circulating levels of thyroid hormones (Fekete et al. 2002). Exposure to phytoestrogens resulted in a slight but significant increase in levels of TRH mRNA, although thyroid hormone levels (total T4 and T3) and core body temperature did not appear to be affected (data not shown).

## Discussion

For this study, we focused our attention on the effects of a soy-rich diet containing high levels of phytoestrogens instead of exposing animals to pure isoflavones such as genistein or diadzein. Although using pure unconjugated isoflavones represents a more controlled experimental setup, using a soy-based diet constitutes a more relevant model of phytoestrogen exposure for humans, cattle, and rodents. Several data sets using different diet formulations of varying isoflavone content have shown that significant reductions in adipose weight correlated with increasing doses of soy-derived phytoestrogens (Lephart et al. 2004b). Ruhlen et al. (Ruhlen RL, Howdeshell KL, Mao J, Taylor JA, Bronson FH, Welshons WV, vom Saal FS, unpublished data), who followed a similar protocol of dietary soy exposition but used a different supplier, also observed reduced adiposity in mice. Finally, dietary supplementation with genistein has been reported to reduce adipose tissue deposition in a dose-dependent manner (Naaz et al. 2003; Penza et al. 2006), indicating that genistein can have pronounced antilipogenic effects and is probably the major soy constituent contributing to this effect.

Estrogens have been shown to influence proliferation and differentiation of adipocyte precursors during development (Anderson et al. 2001). This suggests that phytoestrogens could also influence adipocyte number. Our results indicate that this is not the case: The effects of dietary phytoestrogens reflect a decrease in adipocyte size. These data are consistent with previous work indicating that genistein produces a dose-dependent decrease in adipocyte size (Naaz et al. 2003). Two important questions remain however. First, the relative roles of ER-α and ER-β in mediating the metabolic effects of dietary phytoestrogens need to be ascertained, because both receptors are expressed in adipocytes and hypothalamic neurons (Kuiper et al. 1997; Pedersen et al. 2001). Second, their modes of action need to be determined: Are phytoestrogens acting centrally by modulating hypothalamic function and/or directly by modulating lipogenesis in adipocytes?

### Central effects of dietary phytoestrogens

Feeding behavior and locomotor activity were modified by exposure to phytoestrogens, suggesting that the hypothalamus, a region of the brain playing a critical role in energy balance, may be involved. The expression of genes coding for the appetite-modifying peptides MCH and orexin A was up-regulated in hypothalami of HP mice. It is thus relevant to note that in rodents, orexin A stimulates eating behavior and physical activity (Kotz et al. 2002), and intracerebroventricular administration of MCH causes rapid increase in food intake (Gomori et al. 2003). AgRP is another critical regulator of energy balance that acts as melanocortin receptor antagonist in hypothalamic regions controlling food intake and energy expenditure (Stutz et al. 2005). The significant reduction of *AgRP* gene expression levels together with the physiologic changes associated with phytoestrogen exposure are strikingly reminiscent of the phenotype of animals lacking AgRP: *AgRP* mutant mice display an age-related lean phenotype correlated with increased metabolic rate, lipid utilization, and locomotor activity (Wortley et al. 2005). Similarly, a 50% inhibition of AgRP expression in the hypothalamus using RNA interference is also associated with increased metabolic rate and reduced body weight (Makimura et al. 2002). Both estrogen receptors are present in the hypothalamic nuclei and have been shown to modulate food intake (Liang et al. 2002), locomotor activity (Ogawa et al. 2003), and the expression of orexigenic signals such as AgRP and NPY (Titolo et al. 2006). Interestingly, the specific silencing of ER-α in the ventromedial nucleus of the hypothalamus in female rodents leads to obesity and declines in energy expenditure, voluntary activity, basal metabolic rate, and thermogenesis (Musatov et al. 2007). Whether these phenotypes are associated with changes in the expression of hypothalamic neuropeptides remains unknown. Overall, these findings indicate that dietary phytoestrogens have a central action and may modulate energy homeostasis in part by affecting the expression of hypothalamic neuropeptides such as orexin A, MCH, and AgRP.

### Phytoestrogens as a source of endocrine disruptors in rodent diet

Our results emphasize the importance of monitoring the phytoestrogen content of diets as a parameter of variability in animal experiments. Research laboratories should carefully select diets for which the isoflavone content has been certified by the supplier. In almost all commercially available diets, soy is used as a main source of protein. The levels of conjugated isoflavones contained in rodent diets are variable and depend on the percentage of soy-derived proteins within the commercial diet itself and also in the individual batch of soy protein that has been incorporated in the diet. The variation in isoflavone content depends on the type of soybean, the geographic area of cultivation, and the harvest year. As a consequence, different batches of an “identical” commercial rodent diet may have variable levels of isoflavones that could lead to inconsistant results (vom Saal FS, Thigpen J, Setchell KDR, Heindel J, unpublished data). The effects of phytoestrogens on adipose tissue and energy balance presented here should strongly encourage research laboratories to select diets for which the content in isoflavones has been certified by the supplier.

The most important issue however is whether these results are relevant for humans. Numerous studies suggest that soy-containing food can have beneficial effects on diabetes and obesity [for review see Usui (2006)]. For example, isoflavone consumption reduces human body mass indexes and insulin levels (Goodman-Gruen and Kritz-Silverstein 2001). Comparisons between animal and human studies are difficult because of variations in exposition protocols (e.g., route of administration, composition, dose, duration) and differences in intestinal bacterial metabolism. Thus far, soy-derived phytoestrogens have received prevalent usage because of their benefits in decreasing age-related diseases (e.g., osteoporosis, cardiovascular disease) and hormone-dependent cancers (e.g., prostate and breast cancer); however, as potent endocrine disruptors, they can also pose potential health risks. A legitimate area of concern is dietary phytoestrogens used by women at risk for breast cancer or those already diagnosed with breast cancer. Similarly, soy-based formula used as an alternative to breast milk or cow’s milk may expose infants to estrogenic compounds at a very vulnerable period for estrogenic insult (West et al. 2005). Although critical questions remain in our understanding of the role of soy and its component phytoestrogens in the regulation of energy balance and obesity, it is now clear that these compounds may have more potent effects than originally thought and that they may well represent an effective natural approach to help prevent some of the metabolic abnormalities that are part of the metabolic syndrome.
